# Nimbolide Targeting SIRT1 Protects Against Acetaminophen‐Induced Acute Liver Injury by Regulating Oxidative Stress and Endoplasmic Reticulum Stress

**DOI:** 10.1002/prp2.70120

**Published:** 2025-05-15

**Authors:** Junhui Ba, Yunsen Lin, Jingcong Zhang, Yanhong Wang, Benquan Wu

**Affiliations:** ^1^ Department of Medical Intensive Care Unit Third Affiliated Hospital of Sun Yat‐Sen University Guangzhou China

**Keywords:** APAP, ER stress, nimbolide, SIRT1

## Abstract

Acetaminophen (APAP) is a major cause of acute liver injury (ALI), and N‐acetylcysteine is the only approved detoxification drug. Nimbolide (Nim), which is isolated from the neem tree ( *Azadirachta indica* ), possesses protective properties against multiple diseases, including pancreatitis, autoimmune hepatitis, arthritis, and diabetic cardiomyopathy. Here, we investigated the protective effect of nimbolide on APAP‐induced ALI. Male C57BL/6J mice were used to establish an ALI model via APAP administration (500 mg/kg, i.p.). All the mice received nimbolide (20 mg/kg, i.p.) or a vehicle 2 h before APAP injection. Blood and liver samples were collected at the indicated times. As expected, Nim treatment alleviated APAP‐induced liver injury and inflammation in the mice. Moreover, Nim inhibited APAP‐induced apoptosis by regulating endoplasmic reticulum (ER) stress. We further revealed that Nim improved mitochondrial function and increased Sirtuin 1 (SIRT1) expression. However, the protective effects of Nim were partially blocked by SIRT1 knockdown via siRNA in vitro. Our study revealed that nimbolide alleviated APAP‐induced ALI by inhibiting oxidative stress and ER stress via SIRT1 activation.

## Graphical abstract

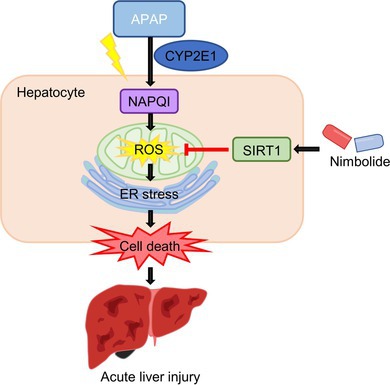

## 1 Introduction

Acetaminophen (APAP) is the most widely used antipyretic and analgesic drug and is dose‐limited because of its potential to induce severe liver injury [1]. APAP overdose is the leading cause of drug‐induced acute liver injury (ALI) worldwide, accounting for more than half of drug‐induced ALI cases in the United States [2]. To date, N‐acetylcysteine (NAC) is the only approved detoxification drug for APAP‐induced hepatotoxicity [3]. However, the clinical use of NAC is limited due to its narrow therapeutic window and several serious side effects [4]. The discovery of a novel intervention to treat APAP‐induced ALI would be of great clinical importance.

Evidence suggests that mitochondria are sites of reactive oxygen species (ROS) formation during APAP overdose. During APAP‐induced ALI, excessive mitochondrial ROS (mtROS) can cause lipid peroxidation and lead to cross‐linking of proteins, DNA, and lipids. These effects trigger mitochondrial dysfunction, leading to cell death [5, 6]. Although various forms of cell death are involved in APAP‐induced ALI, endoplasmic reticulum (ER) stress plays a significant role in driving cell death. ER stress is triggered by the accumulation of unfolded or misfolded proteins in the ER [7]. When this accumulation exceeds the upper limit of ER folding clearance, the unfolded protein response (UPR), an evolutionarily conserved signaling process, is initiated and coordinated via three ER‐transmembrane proteins: activating transcription factor 6 (ATF6), inositol‐requiring transmembrane kinase/endoribonuclease 1α (IRE1α), and protein kinase R‐like ER kinase (PERK) [8]. Increasing evidence suggests that ER stress exacerbates APAP‐induced ALI [5, 9, 10]. Since ER stress can be induced by oxidative stress [11], multiple pharmaceuticals targeting mitochondrial dysfunction have been reported to alleviate ER stress‐induced cell death in APAP‐induced ALI [11, 12].

Sirtuin 1 (SIRT1), a nicotinamide adenine dinucleotide (NAD)‐dependent type III deacetylase, is believed to be involved in various biological functions, including DNA repair, apoptosis, senescence, inflammation, and autophagy [13]. SIRT1 is also involved in controlling the hepatotoxicity of APAP by regulating inflammation and oxidative stress [14]. Furthermore, SIRT1 has been reported to participate in modulating the ER stress response in the heart [15, 16]. However, whether SIRT1 can modulate ER stress to alleviate APAP‐induced ALI remains to be investigated.

Nimbolide (Nim), a type of limonoid extracted from the neem tree, is a highly oxidized tetranortriterpenoid [17]. Previous studies have shown that Nim has various biological properties, including antitumor [18], antibacterial [19], anti‐inflammatory [20] and antioxidative [21] effects. Interestingly, recent studies have shown that Nim exerts protective effects via a SIRT1‐dependent mechanism in disc degeneration [22], acute pancreatitis [23] and chronic pancreatitis [24]. However, the protective effects of Nim against ALI and the underlying molecular mechanisms remain unclear. Given that Nim has a potent effect on the induction of SIRT1, it may be an attractive therapeutic candidate for the treatment of ALI.

In this study, we found that Nim administration markedly improved APAP‐induced ALI in a mouse model. We further demonstrated that Nim‐induced SIRT1 expression significantly alleviated APAP‐induced mitochondrial dysfunction and ER stress. Silencing and inhibition of SIRT1 blocked this beneficial effect of Nim, indicating that Nim prevents APAP‐induced hepatotoxicity in a SIRT1‐dependent manner.

## 2 Materials and Methods

### 2.1 Animals and Treatment

Male C57BL/6J mice (6–8 weeks, 18–22 g) were purchased from GemPharmatech Co. Ltd. (Jiangsu, China) and housed in the Experimental Animal Center of the Third Affiliated Hospital of Sun Yat‐sen University. All the mice were maintained under controlled conditions with a 12 h light/dark cycle and provided with food and water ad libitum. After fasting overnight, the mice were intraperitoneally injected with APAP (500 mg/kg, S1634, Selleck, China) to induce acute liver failure. Nim (20 mg/kg, E0455, Selleck, China) or NAC (500 mg/kg, S5804, Selleck, China) was intraperitoneally injected 2 h before APAP administration, and the control group was treated with PBS. The concentration of Nim used in our study (20 mg/kg) was selected on the basis of the reported literature [25]. Mice were sacrificed at 3 h, 6 h, and 12 h post‐APAP injection. Blood and liver samples were collected for further experiments. All animal experiments were conducted according to protocols approved by the Institutional Animal Care and Use Committee of the Third Affiliated Hospital of Sun Yat‐sen University. All procedures used in the present study were done under the ethical guidelines for the Care and Use of Laboratory Animals.

### 2.2 Measurement of Serum ALT and AST

Blood samples were collected and centrifuged at 1000 × g for 10 min at 4°C. Serum was obtained to analyze the levels of ALT and AST via commercial ELISA kits (Elabscience, Wuhan, China) according to the manufacturer's protocols.

### 2.3 Histological Analysis

Liver samples were fixed in 4% formaldehyde, embedded in paraffin, and cut into 4 μm‐thick sections. After routine dewaxing and hydration, the liver tissue sections were stained with hematoxylin and eosin (H&E). For immunohistochemistry (IHC) staining, after antigen retrieval, the sections were incubated with primary antibodies against F4/80 (#70076, 1:400, Cell Signaling Technology), Ly6G (#ab238132, 1:2000, Abcam), and CHOP (#15204‐1‐AP, 1:200, Proteintech) at 4°C overnight. This was followed by incubation with the corresponding secondary antibodies for 1 h at 37°C. Then, the IHC signals were visualized using a DAB kit. For immunofluorescence (IF) staining, after antigen retrieval, the sections were incubated with primary antibodies against 4‐HNE (#ab46545, 1:400, Abcam) at 4°C overnight, followed by incubation with fluorescent secondary antibodies for 1 h at 37°C. Then, the IF signals were visualized via a fluorescence microscope (Zeiss, Germany).

### 2.4 Total GSH and MDA Analysis

The mouse livers and cells were placed in cold PBS and homogenized on ice. The levels of total GSH and MDA in the homogenates were measured using the corresponding commercial kits (Boxbio, Beijing, China) according to the manufacturer's instructions.

### 2.5 Terminal dUTP Nick‐End Labeling (TUNEL) Apoptosis Assay

DNA fragments were labeled in situ by using a TUNEL apoptotic cell detection kit (Servicebio, Wuhan, China). The tissue slices were stained according to the manufacturer's instructions. TUNEL‐positive cells were counted via ImageJ software.

### 2.6 Western Blotting

Total protein was extracted from liver tissues and cell samples via radioimmunoprecipitation assay (RIPA) buffer at 4°C for 30 min. The lysate was subsequently centrifuged at 12000 × g at 4°C for 20 min, after which the protein in the supernatant was collected. After mixing with 5× loading buffer, the purified protein was denatured at 100°C for 10 min and separated via 10%–12% SDS–PAGE gels. The proteins were subsequently transferred to nitrocellulose (NC) filter membranes and blocked with 5% nonfat milk. The membranes were incubated with primary antibodies overnight at 4°C, followed by incubation with HRP‐conjugated secondary antibodies at room temperature for 1 h. Then the signals were visualized with chemiluminescence reagents and measured via Image J software. The following antibodies were used: Bcl2 (#ab196495, 1:1000, Abcam), cleaved caspase 3 (#19677‐1‐AP, 1:1000, Proteintech), p‐eIF2α (#3398, 1:1000, Cell Signaling Technology), eIF2α (#5324, 1:1000, Cell Signaling Technology), CHOP (#2895, 1:1000, Cell Signaling Technology), PUMA (#98672, 1:1000, Cell Signaling Technology), GAPDH (#5174, 1:4000, Cell Signaling Technology), CYP2E1 (#19937‐1‐AP, 1:2000, Proteintech) and SIRT1 (#9475, 1:1000, Cell Signaling Technology).

### 2.7 Quantitative Real‐Time Polymerase Chain Reaction (qRT–PCR)

Total RNA was extracted from liver tissues via Trizol reagent according to the manufacturer's instructions. The RNA was quantified via a NanoDrop (Thermo Fisher Scientific, CA, USA) and then reverse transcribed into cDNA using a cDNA synthesis kit (Takara, Japan). Quantitative PCR was performed on a PCR detection system (Bio‐Rad, CA, USA) with SYBR Green qPCR mix (Vazyme, Nanjing, China) and gene‐specific primers. Relative gene expression was normalized to that of GAPDH. The primer sequences are shown in Table 1.

**TABLE 1:** Primers for real‐time PCR analysis.

Name	Sequence (5′–3′)
Mouse IL‐1α	
F	CACAACTGTTCGTGAGCGCT
R	TTGGTGTTTCTGGCAACTCCT
Mouse IL‐1β	
F	GCAACTGTTCCTGAACTCAACT
R	ATCTTTTGGGGTCCGTCAACT
Mouse IL‐6	
F	AGGATACCACTCCCAACAGACCT
R	CAAGTGCATCATCGTTGTTCATAC
Mouse TNF‐α	
F	TTCTGTCTACTGAACTTCGGGGTGATCGGTCC
R	GTATGAGATAGCAAATCGGCTGACGGTGTGGG
Mouse ATF4	
F	ATAGAAGAGGTCCGTAAGGCAA
R	CAGCAAACACAGCAACACAAGA
Mouse CHOP	
F	GTTGAAGATGAGCGGGTGG
R	CAAGGTGAAAGGCAGGGAC
Mouse BIP	
F	GCTGGTGTCCTCTCTGGTGAT
R	TGTCTTTTGTTAGGGGTCGTT
Mouse XBP1	
F	AGCAGCAAGTGGTGGATTTG
R	GAGTTTTCTCCCGTAAAAGCTGA
mtDNA	
F	CCCAGCTACTACCATCATTCAAGT
R	GATGGTTTGGGAGATTGGTTGATGT
Mouse GAPDH	
F	GGCTCCCTAGGCCCCTCCTG
R	TCCCAACTCGGCCCCCAACA

### 2.8 Cell Culture, Treatment and Transfection

The murine normal hepatocyte line AML12 was purchased from the China Cell Bank and cultured in Nutrient Mixture F‐12 (DMEM/F‐12) supplemented with 10% fetal bovine serum (Gibco, CA, USA), 5 μg/mL insulin, 5 μg/mL transferrin, 5 ng/mL selenium, and 40 ng/mL dexamethasone. AML12 cells were pretreated with Nim (5 μM) for two hours and then treated with APAP (10 mM) for another 6 h. AML12 cells were transfected with siRNA specifically targeting SIRT1 using Lipofectamine 2000 transfection reagent (Invitrogen, CA, USA) according to the manufacturer's instructions. The sequence (antisense) of the siRNA targeting SIRT1 was (5′–3′) AGUGAGACCAGUAGCACUAAUTT.

### 2.9 Measurement of Mitochondrial DNA (mtDNA) Copy Number

Mitochondrial DNA was extracted from mouse liver tissues using a Genomic DNA Extraction Kit (Qiagen, Venlo, Netherlands). qRT–PCR was used to measure the relative copy number of mtDNA and nuclear DNA. The quantity of mitochondrial DNA was analyzed with mitochondrial DNA‐specific sequences and normalized to the number of nuclear DNA sequences (GAPDH). The primer sequences are listed in Table 1.

### 2.10 Measurement of Liver ATP Content

The levels of ATP in mouse liver were determined using an Enhanced ATP Assay Kit (Beyotime, Shanghai, China) following the manufacturer's instructions.

### 2.11 Detection of Cellular ROS and Mitochondrial ROS

Cellular ROS and mitochondrial ROS were detected by DCFH‐DA (Sigma–Aldrich, MO, USA) and MitoSOX (Invitrogen, CA, USA), respectively. Briefly, the cells were incubated with 10 μm DCFH‐DA or 2.5 μm MitoSOX working solution at 37°C for 30 min in the dark. After the samples were washed with PBS twice, the fluorescence signals were captured via fluorescence microscopy and calculated via ImageJ software.

### 2.12 Statistical Analysis

Statistical analysis was performed using the GraphPad Prism 8.0.2, and the data are expressed as the means ± SDs. Student's *t* test between two groups or one‐way ANOVA between more than two groups was used to calculate the statistical significance. Differences between groups were considered statistically significant at *p* < 0.05. All the statistical data in this work were obtained from at least 3 independent experiments.

## 3 Results

### 3.1 Nim Protects Against APAP‐Induced Liver Injury in Mice

**FIGURE 1:**
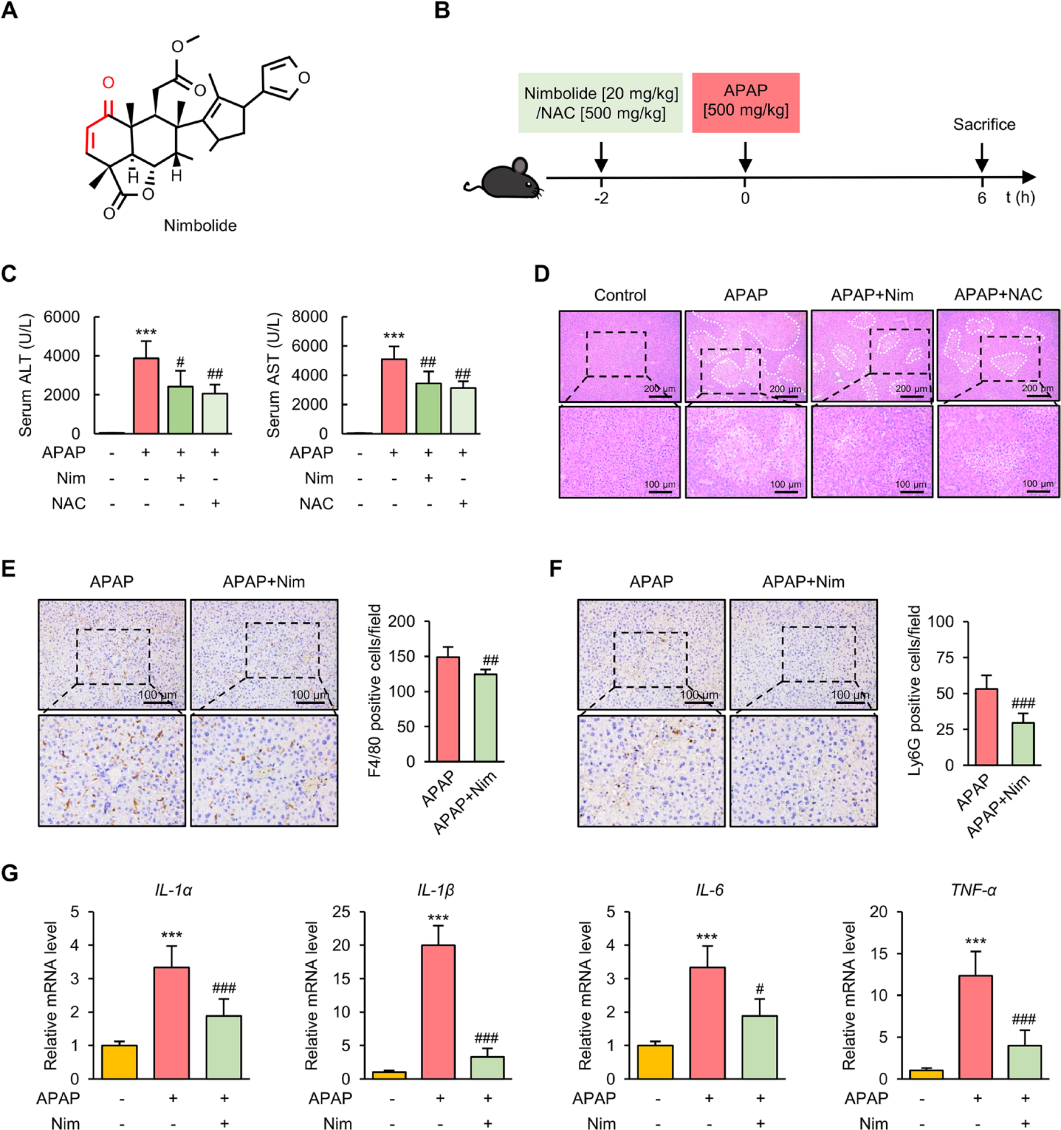
Nim protects against APAP‐induced liver injury and inflammation in mice. (A) The chemical structure of Nimbolide. (B) Schematic diagram of the experimental procedures. (C) Serum ALT and AST levels at 6 h after APAP injection. (D) Representative images of H&E staining in livers of mice at 6 h after APAP injection. (E) Representative images of IHC staining and quantification of positive cells of the liver F4/80 in the indicated mice. (F) Representative images of IHC staining and quantification of positive cells of the liver Ly6G in the indicated mice. (G) *IL‐1α*, *IL‐1β*, *IL‐6*, and *TNF‐α* mRNA levels measured by qRT‐PCR assays. All data were expressed as mean ± SD (*n* = 6 per group) and the statistical differences were analyzed by Student's *t* test or one‐way ANOVA (E and F, Student's *t* test; C and G, one‐way ANOVA). ***p < 0.001 vs. control group; ^#^ *p* < 0.05, ^##^ *p* < 0.01, ^###^ *p* < 0.001 vs. APAP group.

Nimbolide (Figure 1A) possesses a cyclic enone that is potentially cysteine‐reactive. To investigate its role in APAP‐induced liver injury, mice were pretreated with Nim and then administered APAP to induce acute liver injury, as depicted in Figure 1B. As NAC is the only approved detoxification drug, we also included NAC as a positive control treatment group in our animal experiments. First, we measured the levels of serum aminotransferases and analyzed the histopathological changes in the liver tissue of APAP‐treated mice. We found that Nim treatment significantly reduced the serum ALT and AST levels in mice with APAP‐induced liver injury, with therapeutic effects comparable to those of NAC (Figure 1C). APAP overdose destroys hepatocytes and induces necrosis in the centrilobular area within 6 h [26]. Accordingly, H&E staining (Figure 1D) revealed that APAP administration caused massive necrosis in the centrilobular region compared with mice treated with the control vehicle, and both Nim and NAC alleviated the hepatic histopathology and necrotic area of the liver. Damage‐associated molecular patterns (DAMPs) leaked from damaged hepatocytes activate a severe immune response to amplify liver injury in ALI [2]. We next investigated inflammatory cell infiltration in the liver at the indicated time points. We found that Nim significantly reduced the numbers of F4/80+ (macrophages, Figure 1E) and Ly6G+ (neutrophils, Figure 1F) cells in the livers of the APAP‐treated mice. Moreover, Nim inhibited the expression of inflammatory genes, including *IL‐1α*, *IL‐1β*, *IL‐6*, and *TNF‐α* (Figure 1G). Collectively, these results suggest that Nim has a protective role in APAP‐induced acute liver injury and inflammation in mice.

### 3.2 Nim Suppresses APAP‐Induced Hepatocellular Apoptosis in Mice

**FIGURE 2:**
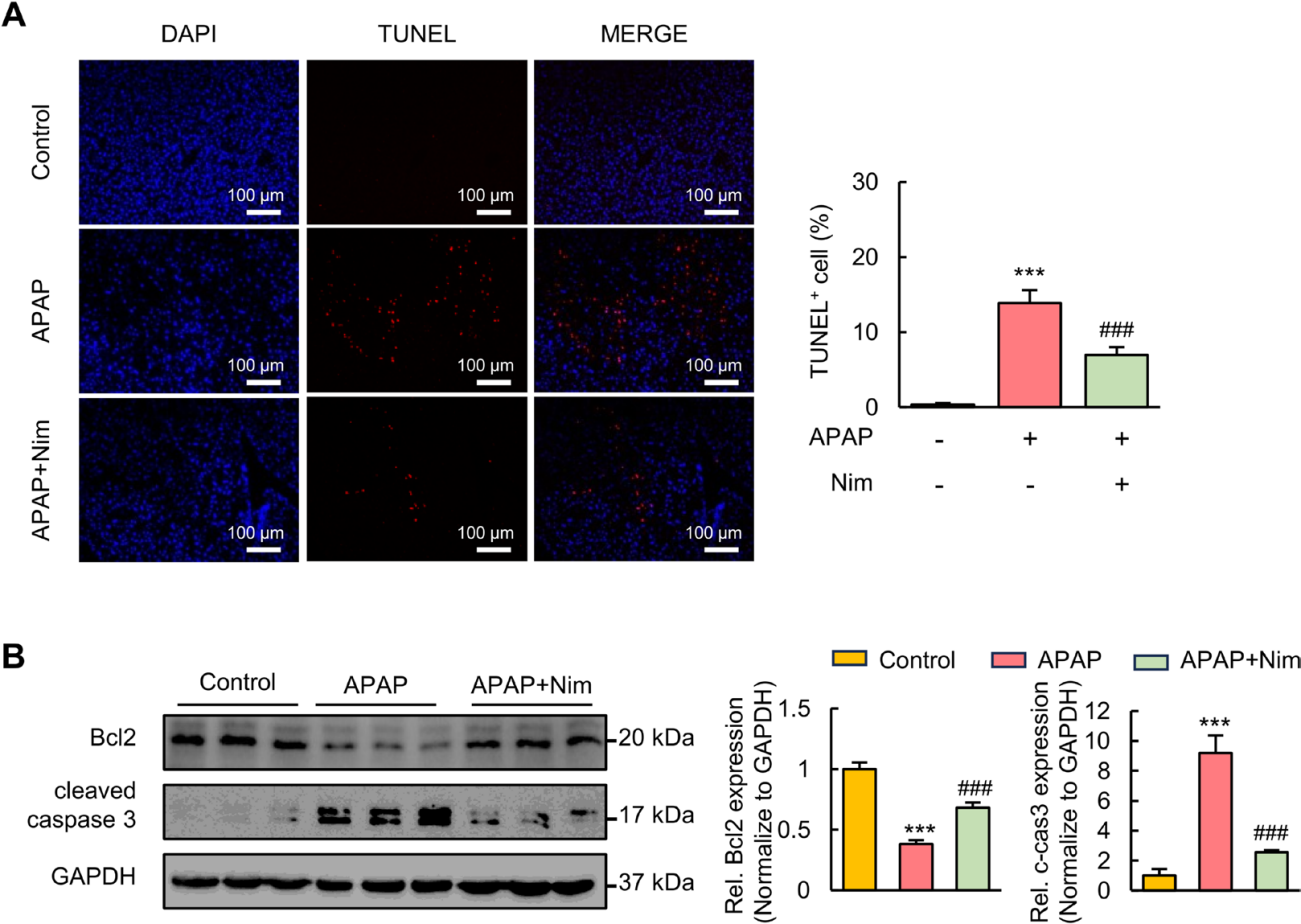
Nim suppresses APAP‐induced hepatocellular apoptosis in mice. (A) Representative images of TUNEL staining and quantification of TUNEL positive cells of the livers in the indicated mice. (B) Protein expressions of Bcl2 and cleaved Caspase 3 analyzed by western blot. All data were expressed as mean ± SD (*n* = 3–6 per group) and the statistical differences were analyzed by one‐way ANOVA. ***p < 0.001 vs. control group; ###*p* < 0.001 vs. APAP group.

During the process of APAP‐induced ALI, hepatocytes suffer massive oxidative stress and severe inflammation, which may lead to cellular apoptosis [27]. As shown in Figure 2A, the TUNEL staining assay revealed that APAP induced a large amount of fragmented DNA in the liver, which emerged from cellular apoptosis. As expected, Nim markedly decreased the number of apoptotic cells in the APAP‐treated group. In addition, the levels of Bcl2 in the APAP‐treated group were lower than those in the control group (Figure 2B). Nim treatment upregulated the expression of Bcl2 in APAP‐treated mice. The levels of cleaved caspase 3 exhibited opposite trends to those of Bcl2 in the livers of the mice. These results showed that Nim alleviated apoptosis induced by APAP administration.

### 3.3 Nim Inhibits Apoptosis by Regulating Endoplasmic Reticulum Stress in APAP‐Treated Mice

**FIGURE 3:**
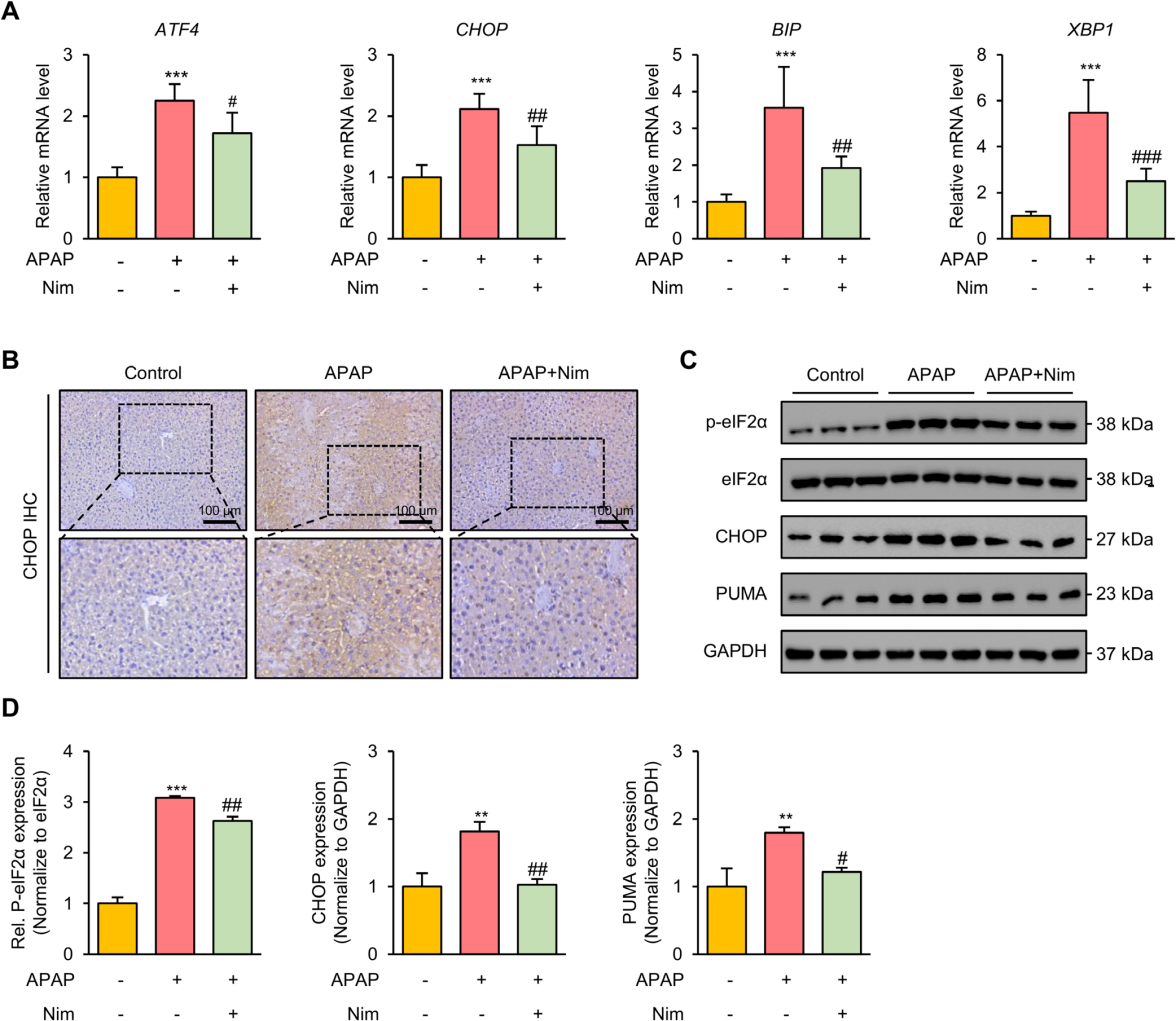
Nim inhibits apoptosis by regulating endoplasmic reticulum stress in APAP‐treated mice. (A) *ATF4*, *CHOP*, *BIP*, and *XBP1* mRNA levels measured by qRT‐PCR assays. (B) Representative images of IHC staining of the liver CHOP in the indicated mice. (C and D) Protein expressions of ER‐stress‐related genes analyzed by western blot. All data were expressed as mean ± SD (*n* = 3–6 per group) and the statistical differences were analyzed by one‐way ANOVA. ***p < 0.001 vs. control group; ^#^ *p* < 0.05, ^##^ *p* < 0.01, ^###^ *p* < 0.001 vs. APAP group.

ER stress‐mediated apoptosis plays a key role in the hepatotoxicity of APAP [28]. We next investigated the expression of ER stress‐associated genes. As shown in Figure 3A, APAP overdose increased the mRNA levels of ATF4, CHOP, BIP, and XBP1, whereas Nim treatment significantly decreased the expression of these genes. CHOP is a critical mediator of APAP‐induced hepatotoxicity and acts as a transcriptional repressor to promote apoptosis [28]. Accordingly, immunohistochemical (IHC) staining revealed that APAP treatment increased the expression of CHOP in both the cytoplasm and nucleus, which was significantly reduced by Nim (Figure 3B). In addition, PUMA, a Bcl‐2 homology 3 (BH3)‐only member of the Bcl‐2 family, is transcriptionally activated by the eIF2α‐ATF4‐CHOP axis and is essential for ER stress‐related apoptosis [29]. We found that the phosphorylation level of eIF2α and the protein expression of CHOP and PUMA were also increased by APAP overdose, but significantly reduced by Nim treatment (Figure 3C,D). These results indicated that Nim might inhibit APAP‐induced apoptosis by mediating ER stress signaling.

### 3.4 Nim Inhibits APAP‐Induced Mitochondrial Dysfunction and Oxidative Stress In Vivo and In Vitro

**FIGURE 4:**
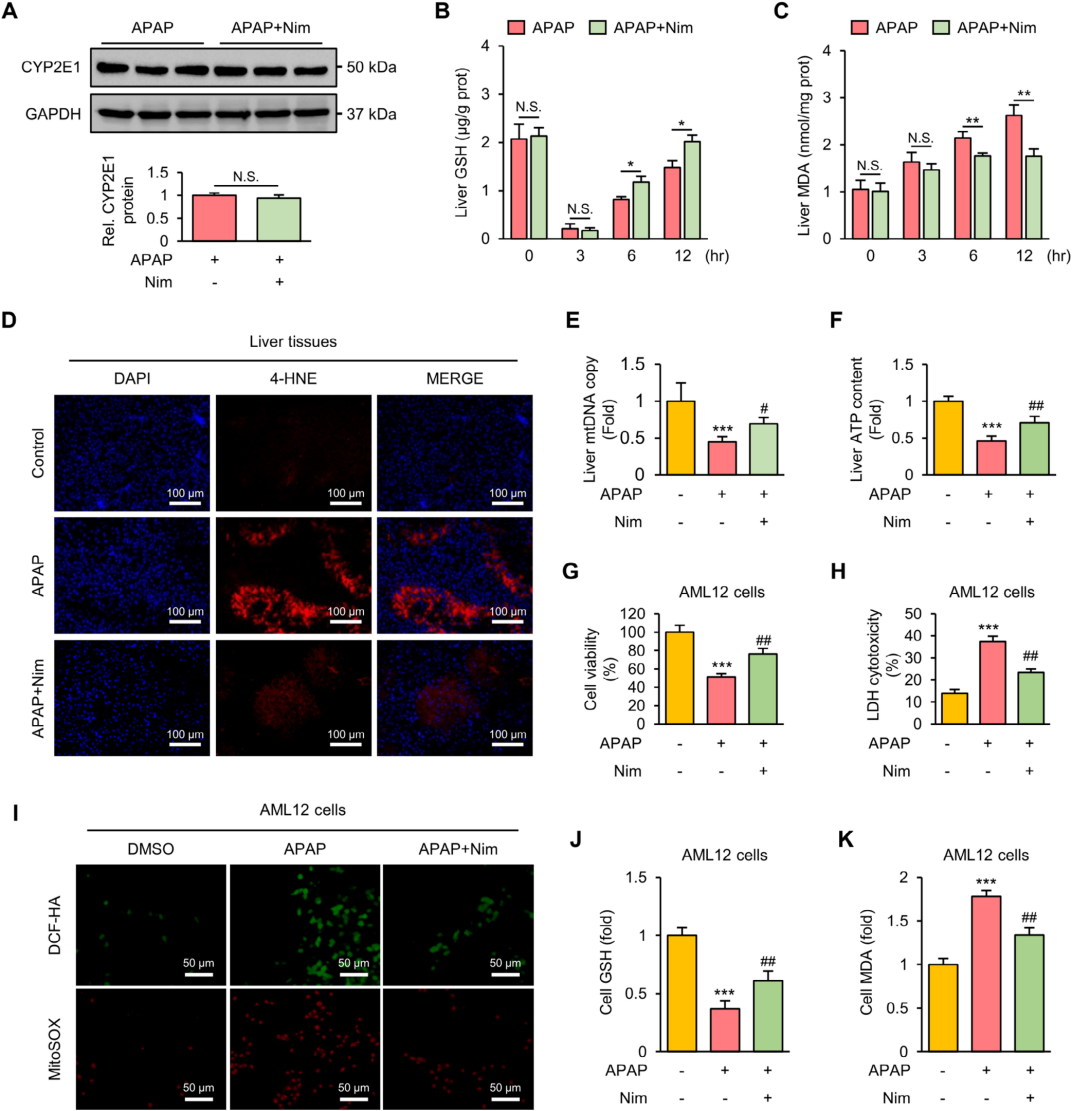
Nim inhibits APAP‐induced mitochondrial dysfunction and oxidative stress in vivo and in vitro. (A) Protein expression of CYP2E1 in mice liver at 3 h after APAP injection. (B) Liver GSH contents in mice liver at 0, 3, 6, and 12 h after APAP injection. (C) Liver MDA contents in mice liver at 0, 3, 6, and 12 h after APAP injection. (D) Representative images of IF staining of the liver 4‐HNE in the indicated mice at 6 h after APAP injection. (E) Relative mitochondrial DNA copy number in mice liver at 6 h after APAP injection. (F) Relative ATP content in mice liver at 6 h after APAP injection. (G) Cell viability of AML12 cells in indicated groups. (H) LDH cytotoxicity of AML12 cells in indicated groups. (I) Representative images of DCF‐HA and MitoSOX staining of AML12 cells in indicated groups. (J) Relative cell GSH contents of AML12 cells in indicated groups. (K) Relative cell MDA contents of AML12 cells in indicated groups. Data in (A–C) were expressed as mean ± SD (*n* = 3 per group) and the statistical differences were analyzed by Student's *t* test. *p < 0.05, **p < 0.01, ***p < 0.001 and N.S., not significant. Data in (E–I) were expressed as mean ± SD (E and F, *n* = 6 per group; G–K, *n* = 3 per group) and the statistical differences were analyzed by one‐way ANOVA. ***p < 0.001 vs. control group; ^#^ *p* < 0.05, ^###^ *p* < 0.001 vs. APAP group.

Mitochondrial dysfunction and oxidative stress are important mechanisms that trigger ER stress [30]. We first examined the expression of CYP2E1, which is the major metabolic enzyme for APAP. Nim did not affect the expression of CYP2E1 at 3 h post‐APAP (Figure 4A), indicating that Nim did not inhibit the metabolic activity of APAP. Consistent with this result, GSH depletion at 3 h was not improved by Nim (Figure 4B). However, Nim significantly accelerated the rate of GSH recovery and reduced hepatic malondialdehyde (MDA) accumulation (Figure 4C). 4‐Hydroxynonenal, like MDA, is another marker of lipid peroxidation. As shown in Figure 4D, APAP increased the area of 4‐HNE‐positive signal in the liver at 6 h post injection compared with that in the control group. Nim markedly decreased the expression of 4‐HNE in APAP‐treated mice. We further examined mitochondrial function by measuring the mtDNA copy number and ATP content in the livers of the indicated mice. Both the mtDNA copy number (Figure 4E) and ATP content (Figure 4F) were decreased in the liver by APAP treatment, indicating mitochondrial dysfunction. However, Nim increased the mtDNA copy number and ATP content to some degree.

We also investigated the antioxidative role of Nim in vitro. Firstly, we performed a CCK8 assay to determine the effect of nimbolide on the viability of AML12 cells, a normal murine hepatic cell line. Our results revealed that nimbolide had minimal effects on the viability of AML12 cells at concentrations up to 5 μM after 24 h of treatment (Figure S1A). Previous studies have shown that 10 mM of APAP can induce cytotoxicity and oxidative stress in hepatic cells [5, 31]. AML12 cells were pretreated with Nim (5 μM) for 2 h and then treated with APAP (10 mM) for another 6 h. Cell viability was significantly reduced by APAP treatment but was restored by Nim pretreatment (Figure 4G). Similarly, Nim pretreatment protected AML12 cells against APAP‐induced cell death, as shown by the decrease in LDH release (Figure 4H). Cellular ROS and mitochondrial ROS were measured by DCF‐HA staining and MitoSOX staining, respectively. APAP significantly increased the levels of both cellular and mitochondrial ROS in AML12 cells (Figure 4I), whereas Nim decreased these levels. Moreover, Nim significantly increased the GSH content in APAP‐treated AML12 cells (Figure 4J) and decreased the MDA content in APAP‐treated AML12 cells (Figure 4K). Collectively, these results indicate that Nim alleviates APAP‐induced mitochondrial dysfunction and oxidative stress.

### 3.5 Nim Protects Against APAP‐Induced Cell Death by Upregulating SIRT1 Expression

**FIGURE 5:**
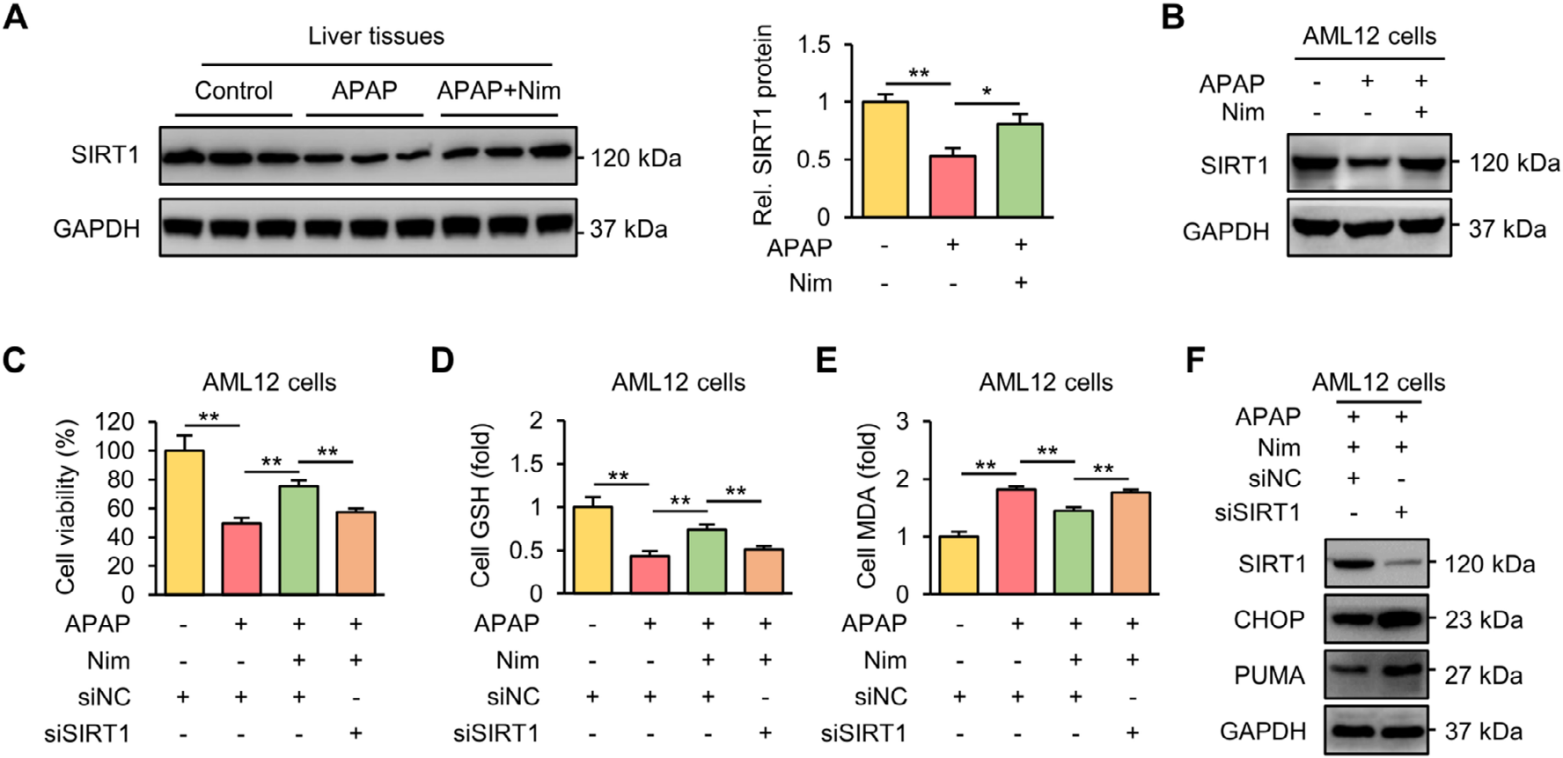
Nim protects against APAP‐induced cell death via upregulating SIRT1 expression. (A) Protein expression of SIRT1 in mice liver is analyzed by western blot. (B) Protein expression of SIRT1 in AML12 is analyzed by western blot. (C) Cell viability of AML12 cells in indicated groups. (D) Relative cell GSH contents of AML12 cells in indicated groups. (E) Relative cell MDA contents of AML12 cells in indicated groups. (F) Protein expression of SIRT1, CHOP, and PUMA in AML12 cells analyzed by western blot. All data were expressed as mean ± SD (*n* = 3 per group) and the statistical differences were analyzed by one‐way ANOVA. *p < 0.05, **p < 0.01.

Recently, SIRT1 has been shown to be a candidate target for Nim [22]. To verify whether Nim protects against APAP‐induced ALI via the activation of SIRT1, we investigated the expression of SIRT1 in the livers of the mice. We found that the protein level of SIRT1 was significantly decreased by APAP overdose but was increased by Nim treatment (Figure 5A). Similarly, APAP inhibited the expression of SIRT1 in AML12 cells, which was increased by Nim pretreatment (Figure 5B). Furthermore, to confirm that the protective role of Nim is mediated by SIRT1, we inhibited the expression of SIRT1 by siRNA in AML12 cells. As mentioned above, APAP stimulation decreased the cell viability (Figure 5C) and GSH content (Figure 5D) of AML12 cells and increased the MDA content (Figure 5E) in AML12 cells. These effects were partially reversed by Nim pretreatment but abolished by the knockdown of SIRT1, suggesting that SIRT1 is essential for the protective effect of Nim. Consistently, the knockdown of SIRT1 significantly reversed the effects of Nim on the expression of the ER stress‐related protein CHOP and its downstream protein PUMA after Nim/APAP cotreatment in AML12 cells (Figure 5F). These data indicate that SIRT1 is involved in Nim‐mediated protection against APAP‐induced oxidative stress, ER stress, and cell death.

## 4 Discussion

**FIGURE 6:**
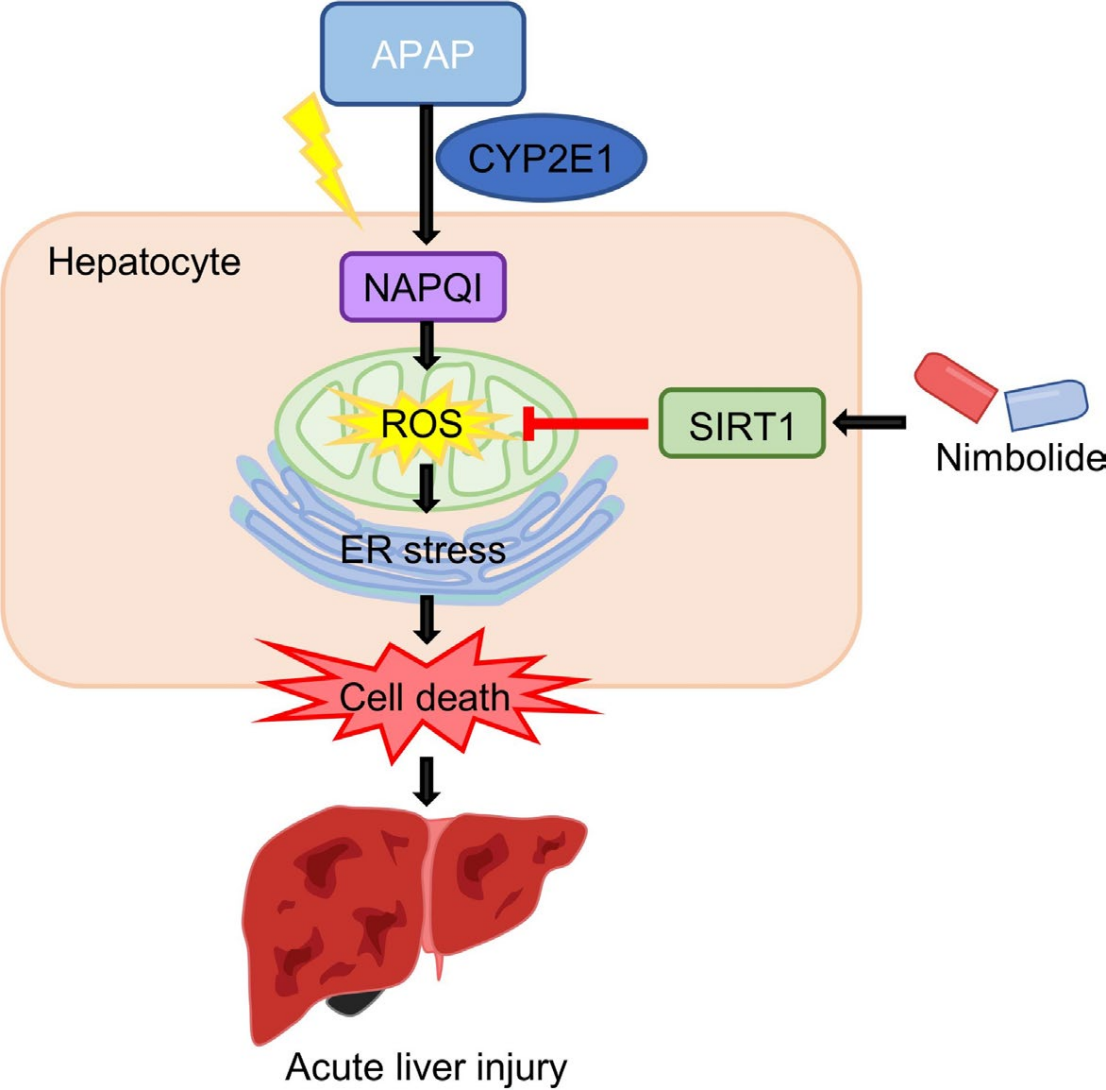
Schematic diagram showing how Nim protects against APAP‐induced acute liver injury.

In this study, we found that Nim protected against APAP‐induced acute liver injury. Moreover, Nim treatment attenuated the inflammatory response and ER stress‐related apoptosis induced by APAP hepatotoxicity. We also found that Nim improved APAP‐induced mitochondrial dysfunction by decreasing ROS levels. These data suggest that Nim is a potential and effective therapeutic option for targeting oxidative stress in APAP‐induced ALI. A schematic diagram showing how Nim protects against APAP‐induced ALI is presented in Figure 6.

To date, APAP overdose remains the most common cause of drug‐induced ALI worldwide. Under normal conditions, APAP is metabolized in the liver by CYP2E1 to generate NAPQI, which is then detoxified by hepatic GSH. Once APAP is overused, excessive NAPQI results in the depletion of hepatic GSH, leading to poor antioxidant activities and massive production of ROS [32]. Excessive oxidative stress in APAP‐induced liver injury causes the activation of the inflammatory response via DAMPs, which are related to mitochondrial DNA, fragmented nuclear DNA, ATP, uric acid, and other contents that are released from damaged cells [33, 34]. APAP‐induced liver injury leads to the induction of inflammatory cytokines (such as IL‐1β, IL‐6, and TNF‐α), which further exacerbate liver damage [35]. Our results showed that Nim protected against APAP‐induced liver injury in mice. Accordingly, the ALT and AST serum levels and necrotic areas of the liver were improved by Nim in APAP‐treated mice. Moreover, pretreatment significantly suppressed inflammatory cell infiltration in the liver induced by APAP. In addition, the mRNA expression levels of *IL‐1α*, *IL‐1β*, *IL‐6*, and *TNF‐α* were also significantly reduced by Nim in APAP‐treated mice.

Previous studies have demonstrated that ER stress is activated by severe oxidative stress in early APAP hepatotoxicity, leading to cell death and liver injury [9, 36]. ER stress is generally transduced by three sensors (PERK, IRE1, and ATF6) that are located in the ER and undergo activation under pathological conditions. Dotan et al. [28] found that the PERK‐eIF2α‐CHOP signaling cascade is involved in APAP‐induced hepatotoxicity. Briefly, eIF2α is phosphorylated by activated PERK and then promotes the translation of ATF4, leading to the transcription of CCAAT‐enhancer‐binding protein homologous protein (CHOP) in a sequential manner. CHOP suppresses the expression of antiapoptotic genes and activates proapoptotic genes, which mediate ER stress‐related apoptosis under various conditions [37, 38]. In this study, we found that Nim alleviated APAP‐induced ER stress‐associated proteins, including CHOP and PUMA, and decreased cell apoptosis in the liver. These results indicate that Nim might protect against APAP‐induced apoptosis by regulating the ER stress signaling pathway.

Mitochondria are widely known as the major source of ROS and the predominant intracellular organelles targeted by NAPQI, as mitochondrial dysfunction occurs at a very early stage during APAP‐induced liver injury [39]. Moreover, mitochondrial oxidative stress and mitochondrial dysfunction are upstream triggers of ER stress, which, in turn, exacerbate mitochondrial dysfunction [40]. Nim reportedly plays a key role in protecting against various oxidative stress‐related diseases [21, 41, 42]. SIRT1 is a sirtuin that regulates cellular function by deacetylating substrates [43]. Nim can strongly bind to SIRT1 and interact with the active sites ASN226 and THR209 to increase its deacetylation activity [22]. In addition, Nim has been suggested to exert strong anti‐inflammatory and antiapoptotic effects on cerulein‐induced acute pancreatitis via SIRT1 activation [23]. However, the correlation between SIRT1 and the protective role of Nim in APAP‐induced ALI has not been investigated.

Our study has several limitations. First, we did not conduct pharmacokinetic studies of Nim in vivo. Future research could address this gap to better understand the actions of Nim. Additionally, although our study demonstrated that Nim protects against APAP‐induced liver injury via SIRT1 activation, we did not provide direct evidence of the interaction between Nim and SIRT1. Future studies could employ techniques such as the cellular thermal shift assay (CETSA) or surface plasmon resonance (SPR) to validate if SIRT1 is a direct binding target of Nim. Third, our study lacks in vivo validation using SIRT1 knockout models to confirm the target specificity of Nim.

In summary, the results of the present study indicate that Nim has a potential therapeutic effect on APAP‐induced acute liver injury, which can be attributed to its ability to target SIRT1. Nim inhibits oxidative stress to improve mitochondrial dysfunction and ultimately reduces the ER stress‐related apoptosis and inflammatory response induced by APAP. Further research is needed to evaluate the potential value of nimbolide in further clinical applications.

## Author Contributions

J.B. and B.W. designed the experiments. J.B. performed the experiments. Y.L. and J.Z. helped collect and analyze the data. B.W. supervised the experiments and edited the manuscript. All authors approved the final version of the manuscript.

## Ethics Statement

All animal experiments were conducted according to protocols approved by the Institutional Animal Care and Use Committee of the Third Affiliated Hospital of Sun Yat‐sen University.

## Conflicts of Interest

The authors declare no conflicts of interest.

## Supporting information


**Figure S1.** The effect of Nim on the viability of AML12 cells.

## Data Availability

The authors declare that all the data supporting the findings of this paper are available from the corresponding author upon reasonable request.

## References

[1] S. J. Patel , J. M. Milwid , K. R. King , et al., “Gap Junction Inhibition Prevents Drug‐Induced Liver Toxicity and Fulminant Hepatic Failure,” Nature Biotechnology 30, no. 2 (2012): 179–183. 10.1038/nbt.2089 PMC3609650 22252509

[2] A. M. Araujo , M. M. Antunes , M. S. Mattos , et al., “Liver Immune Cells Release Type 1 Interferon due to DNA Sensing and Amplify Liver Injury From Acetaminophen Overdose,” Cells 7, no. 8 (2018): 88, 10.3390/cells7080088. 30060463 PMC6115735

[3] H. Jaeschke , J. Y. Akakpo , D. S. Umbaugh , and A. Ramachandran , “Novel Therapeutic Approaches Against Acetaminophen‐Induced Liver Injury and Acute Liver Failure,” Toxicological Sciences 174, no. 2 (2020): 159–167. 31926003 10.1093/toxsci/kfaa002 PMC7098369

[4] R. Yang , K. Miki , X. He , M. E. Killeen , and M. P. Fink , “Prolonged Treatment With N‐Acetylcystine Delays Liver Recovery From Acetaminophen Hepatotoxicity,” Critical Care 13, no. 2 (2009): R55. 19358737 10.1186/cc7782 PMC2689502

[5] L. Li , H. Wang , J. Zhang , et al., “SPHK1 Deficiency Protects Mice From Acetaminophen‐Induced ER Stress and Mitochondrial Permeability Transition,” Cell Death and Differentiation 27, no. 6 (2020): 1924–1937. 31827236 10.1038/s41418-019-0471-x PMC7244772

[6] P. J. Ferret , R. Hammoud , M. Tulliez , et al., “Detoxification of Reactive Oxygen Species by a Nonpeptidyl Mimic of Superoxide Dismutase Cures Acetaminophen‐Induced Acute Liver Failure in the Mouse,” Hepatology 33, no. 5 (2001): 1173–1180. 11343246 10.1053/jhep.2001.24267

[7] C. Hetz , “The Unfolded Protein Response: Controlling Cell Fate Decisions Under ER Stress and Beyond,” Nature Reviews. Molecular Cell Biology 13, no. 2 (2012): 89–102. 22251901 10.1038/nrm3270

[8] C. Hetz , K. Zhang , and R. J. Kaufman , “Mechanisms, Regulation and Functions of the Unfolded Protein Response,” Nature Reviews. Molecular Cell Biology 21, no. 8 (2020): 421–438. 32457508 10.1038/s41580-020-0250-z PMC8867924

[9] S. Torres , A. Baulies , N. Insausti‐Urkia , et al., “Endoplasmic Reticulum Stress‐Induced Upregulation of STARD1 Promotes Acetaminophen‐Induced Acute Liver Failure,” Gastroenterology 157, no. 2 (2019): 552–568. 31029706 10.1053/j.gastro.2019.04.023

[10] H. Ye , C. Chen , H. Wu , et al., “Genetic and Pharmacological Inhibition of XBP1 Protects Against APAP Hepatotoxicity Through the Activation of Autophagy,” Cell Death & Disease 13, no. 2 (2022): 143. 35145060 10.1038/s41419-022-04580-8 PMC8831621

[11] X. Zhang , W. Xiong , L. L. Chen , J. Q. Huang , and X. G. Lei , “Selenoprotein V Protects Against Endoplasmic Reticulum Stress and Oxidative Injury Induced by Pro‐Oxidants,” Free Radical Biology & Medicine 160 (2020): 670–679. 32846216 10.1016/j.freeradbiomed.2020.08.011

[12] Y. Yang , M. Liu , T. Zhao , et al., “Epigallocatechin‐3‐Gallate Mo Nanoparticles (EGM NPs) Efficiently Treat Liver Injury by Strongly Reducing Oxidative Stress, Inflammation and Endoplasmic Reticulum Stress,” Frontiers in Pharmacology 13 (2022): 1039558. 36278211 10.3389/fphar.2022.1039558 PMC9585210

[13] B. Lian , J. Zhang , X. Yin , et al., “SIRT1 Improves Lactate Homeostasis in the Brain to Alleviate Parkinsonism via Deacetylation and Inhibition of PKM2,” Cell Reports Medicine 5 (2024): 101684. 39128469 10.1016/j.xcrm.2024.101684 PMC11384727

[14] P. Rada , V. Pardo , M. A. Mobasher , et al., “SIRT1 Controls Acetaminophen Hepatotoxicity by Modulating Inflammation and Oxidative Stress,” Antioxidants & Redox Signaling 28, no. 13 (2018): 1187–1208. 29084443 10.1089/ars.2017.7373 PMC9545809

[15] A. Prola , J. Pires Da Silva , A. Guilbert , et al., “SIRT1 Protects the Heart From ER Stress‐Induced Cell Death Through eIF2α Deacetylation,” Cell Death and Differentiation 24, no. 2 (2017): 343–356. 27911441 10.1038/cdd.2016.138 PMC5299716

[16] J. Pires Da Silva , K. Monceaux , A. Guilbert , et al., “SIRT1 Protects the Heart From ER Stress‐Induced Injury by Promoting eEF2K/eEF2‐Dependent Autophagy,” Cells 9, no. 2 (2020): 426. 32059483 10.3390/cells9020426 PMC7072417

[17] S. C. Gupta , S. Prasad , D. R. Sethumadhavan , M. S. Nair , Y. Y. Mo , and B. B. Aggarwal , “Nimbolide, a Limonoid Triterpene, Inhibits Growth of Human Colorectal Cancer Xenografts by Suppressing the Proinflammatory Microenvironment,” Clinical Cancer Research 19, no. 16 (2013): 4465–4476. 23766363 10.1158/1078-0432.CCR-13-0080 PMC4220790

[18] J. N. Spradlin , X. Hu , C. C. Ward , et al., “Harnessing the Anti‐Cancer Natural Product Nimbolide for Targeted Protein Degradation,” Nature Chemical Biology 15, no. 7 (2019): 747–755. 31209351 10.1038/s41589-019-0304-8 PMC6592714

[19] S. Navinraj , N. M. Boopathi , V. Balasubramani , et al., “Molecular Docking of Nimbolide Extracted From Leaves of *Azadirachta indica* With Protein Targets to Confirm the Antifungal, Antibacterial and Insecticidal Activity,” Indian Journal of Microbiology 63, no. 4 (2023): 494–512. 38031617 10.1007/s12088-023-01104-6 PMC10682360

[20] J. Y. Seo , C. Lee , S. W. Hwang , J. Chun , J. P. Im , and J. S. Kim , “Nimbolide Inhibits Nuclear Factor‐КB Pathway in Intestinal Epithelial Cells and Macrophages and Alleviates Experimental Colitis in Mice,” Phytotherapy Research 30, no. 10 (2016): 1605–1614. 10.1002/ptr.5657 27270592

[21] Y. Ma , S. Xu , J. Meng , and L. Li , “Protective Effect of Nimbolide Against Streptozotocin Induced Gestational Diabetes Mellitus in Rats via Alteration of Inflammatory Reaction, Oxidative Stress, and Gut Microbiota,” Environmental Toxicology 37, no. 6 (2022): 1382–1393. 35212444 10.1002/tox.23491

[22] Y. Teng , Y. Huang , H. Yu , et al., “Nimbolide Targeting SIRT1 Mitigates Intervertebral Disc Degeneration by Reprogramming Cholesterol Metabolism and Inhibiting Inflammatory Signaling,” Acta Pharmaceutica Sinica B 13, no. 5 (2023): 2269–2280. 37250166 10.1016/j.apsb.2023.02.018 PMC10213799

[23] S. Bansod and C. Godugu , “Nimbolide Ameliorates Pancreatic Inflammation and Apoptosis by Modulating NF‐κB/SIRT1 and Apoptosis Signaling in Acute Pancreatitis Model,” International Immunopharmacology 90 (2021): 107246. 33310297 10.1016/j.intimp.2020.107246

[24] S. Bansod , M. Aslam Saifi , A. Khurana , and C. Godugu , “Nimbolide Abrogates Cerulein‐Induced Chronic Pancreatitis by Modulating β‐Catenin/Smad in a Sirtuin‐Dependent Way,” Pharmacological Research 156 (2020): 104756. 32194177 10.1016/j.phrs.2020.104756

[25] H. Zhang , X. Zhao , W. Wei , and C. Shen , “Nimbolide Protects Against Diabetic Cardiomyopathy by Regulating Endoplasmic Reticulum Stress and Mitochondrial Function via the Akt/mTOR Pathway,” Tissue & Cell 90 (2024): 102478. 39053131 10.1016/j.tice.2024.102478

[26] J. A. Hinson , D. W. Roberts , and L. P. James , “Mechanisms of Acetaminophen‐Induced Liver Necrosis,” Handbook of Experimental Pharmacology 196 (2010): 369–405. 10.1007/978-3-642-00663-0_12 PMC2836803 20020268

[27] D. Chen , H. M. Ni , L. Wang , et al., “p53 Up‐Regulated Modulator of Apoptosis Induction Mediates Acetaminophen‐Induced Necrosis and Liver Injury in Mice,” Hepatology 69, no. 5 (2019): 2164–2179. 30552702 10.1002/hep.30422 PMC6461480

[28] D. Uzi , L. Barda , V. Scaiewicz , et al., “CHOP Is a Critical Regulator of Acetaminophen‐Induced Hepatotoxicity,” Journal of Hepatology 59, no. 3 (2013): 495–503. 23665281 10.1016/j.jhep.2013.04.024

[29] Z. Galehdar , P. Swan , B. Fuerth , S. M. Callaghan , D. S. Park , and S. P. Cregan , “Neuronal Apoptosis Induced by Endoplasmic Reticulum Stress Is Regulated by ATF4‐CHOP‐Mediated Induction of the Bcl‐2 Homology 3‐Only Member PUMA,” Journal of Neuroscience 30, no. 50 (2010): 16938–16948. 21159964 10.1523/JNEUROSCI.1598-10.2010 PMC6634926

[30] L. Avalle , A. Camporeale , G. Morciano , et al., “STAT3 Localizes to the ER, Acting as a Gatekeeper for ER‐Mitochondrion Ca2+ Fluxes and Apoptotic Responses,” Cell Death and Differentiation 26, no. 5 (2019): 932–942. 30042492 10.1038/s41418-018-0171-y PMC6214529

[31] W. Hong , X. Zeng , H. Wang , et al., “PGC‐1α Loss Promotes Mitochondrial Protein Lactylation in Acetaminophen‐Induced Liver Injury via the LDHB‐Lactate Axis,” Pharmacological Research 205 (2024): 107228. 38810904 10.1016/j.phrs.2024.107228

[32] H. Jaeschke , A. Ramachandran , X. Chao , and W. X. Ding , “Emerging and Established Modes of Cell Death During Acetaminophen‐Induced Liver Injury,” Archives of Toxicology 93, no. 12 (2019): 3491–3502. 31641808 10.1007/s00204-019-02597-1 PMC6891214

[33] M. R. McGill , M. R. Sharpe , C. D. Williams , M. Taha , S. C. Curry , and H. Jaeschke , “The Mechanism Underlying Acetaminophen‐Induced Hepatotoxicity in Humans and Mice Involves Mitochondrial Damage and Nuclear DNA Fragmentation,” Journal of Clinical Investigation 122, no. 4 (2012): 1574–1583. 22378043 10.1172/JCI59755 PMC3314460

[34] M. R. McGill , V. S. Staggs , M. R. Sharpe , W. M. Lee , and H. Jaeschke , “Serum Mitochondrial Biomarkers and Damage‐Associated Molecular Patterns Are Higher in Acetaminophen Overdose Patients With Poor Outcome,” Hepatology 60, no. 4 (2014): 1336–1345, 10.1002/hep.27265. 24923598 PMC4174728

[35] S. Raevens , S. Van Campenhout , P. J. Debacker , et al., “Combination of Sivelestat and N‐Acetylcysteine Alleviates the Inflammatory Response and Exceeds Standard Treatment for Acetaminophen‐Induced Liver Injury,” Journal of Leukocyte Biology 107, no. 2 (2020): 341–355. 31841237 10.1002/JLB.5A1119-279R

[36] G. Yang , L. Zhang , L. Ma , et al., “Glycyrrhetinic Acid Prevents Acetaminophen‐Induced Acute Liver Injury via the Inhibition of CYP2E1 Expression and HMGB1‐TLR4 Signal Activation in Mice,” International Immunopharmacology 50 (2017): 186–193. 28668488 10.1016/j.intimp.2017.06.027

[37] C. Ji , R. Mehrian‐Shai , C. Chan , Y. H. Hsu , and N. Kaplowitz , “Role of CHOP in Hepatic Apoptosis in the Murine Model of Intragastric Ethanol Feeding,” Alcoholism, Clinical and Experimental Research 29, no. 8 (2005): 1496–1503. 16131858 10.1097/01.alc.0000174691.03751.11 PMC1432051

[38] N. Tamaki , E. Hatano , K. Taura , et al., “CHOP Deficiency Attenuates Cholestasis‐Induced Liver Fibrosis by Reduction of Hepatocyte Injury,” American Journal of Physiology. Gastrointestinal and Liver Physiology 294, no. 2 (2008): G498–G505. 18174271 10.1152/ajpgi.00482.2007

[39] P. J. Donnelly , R. M. Walker , and W. J. Racz , “Inhibition of Mitochondrial Respiration In Vivo Is an Early Event in Acetaminophen‐Induced Hepatotoxicity,” Archives of Toxicology 68, no. 2 (1994): 110–118. 8179480 10.1007/s002040050043

[40] A. P. Arruda , B. M. Pers , G. Parlakgül , E. Güney , K. Inouye , and G. S. Hotamisligil , “Chronic Enrichment of Hepatic Endoplasmic Reticulum‐Mitochondria Contact Leads to Mitochondrial Dysfunction in Obesity,” Nature Medicine 20, no. 12 (2014): 1427–1435. 10.1038/nm.3735 PMC4412031 25419710

[41] V. Pooladanda , S. Thatikonda , O. Sunnapu , et al., “iRGD Conjugated Nimbolide Liposomes Protect Against Endotoxin Induced Acute Respiratory Distress Syndrome,” Nanomedicine 33 (2021): 102351. 33418136 10.1016/j.nano.2020.102351 PMC7833751

[42] V. Pooladanda , S. Thatikonda , S. Bale , et al., “Nimbolide Protects Against Endotoxin‐Induced Acute Respiratory Distress Syndrome by Inhibiting TNF‐α Mediated NF‐κB and HDAC‐3 Nuclear Translocation,” Cell Death & Disease 10, no. 2 (2019): 81. 30692512 10.1038/s41419-018-1247-9 PMC6349848

[43] B. Ponugoti , D. H. Kim , Z. Xiao , et al., “SIRT1 Deacetylates and Inhibits SREBP‐1C Activity in Regulation of Hepatic Lipid Metabolism,” Journal of Biological Chemistry 285, no. 44 (2010): 33959–33970. 20817729 10.1074/jbc.M110.122978 PMC2962496

