# Supplementary material for: Nimbolide Targeting SIRT1 Protects Against Acetaminophen‐Induced Acute Liver Injury by Regulating Oxidative Stress and Endoplasmic Reticulum Stress
Source: Pharmacol Res Perspect. 2025 May 15;13(3):e70120. doi: 10.1002/prp2.70120 (PMC12081326; doi:10.1002/prp2.70120)
Supplement: Supplementary file 1 — Figure S1. The effect of Nim on the viability of AML12 cells. [file PRP2-13-e70120-s001.docx]

Supplementary Materials for

**Nimbolide targeting SIRT1 protects against acetaminophen-induced acute liver injury by regulating oxidative stress and endoplasmic reticulum stress**

Junhui Ba^1,2^, Yunsen Lin^1,2^, Jingcong Zhang^1^, Yanhong Wang^1, *^, Benquan Wu^1,^

^1^Department of Medical Intensive Care Unit, Third Affiliated Hospital of Sun Yat-sen University, 510630 Guangzhou, China.

^2^These authors contributed equally.

^*^Correspondence:

[wyh963@126.com](mailto:wyh963@126.com) (Yanhong Wang), zswubq@163.com (Benquan Wu)


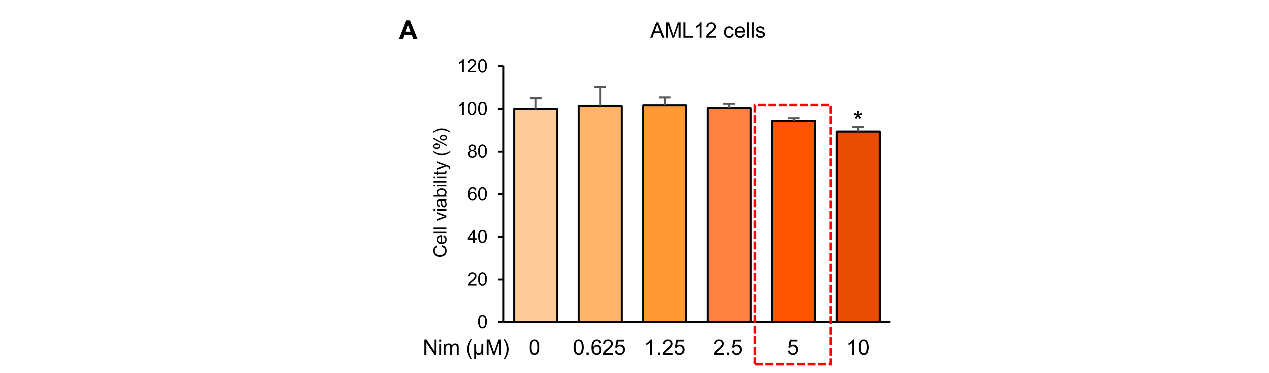


**Figure S1. The effect of Nim on the viability of AML12 cells.**

(A) Cell viability of AML12 cells treated with different concentrations of Nim for 24 h was assessed by CCK8 assay. Data were expressed as mean ± SD (n = 3 per group) and the statistical differences were analyzed by one-way ANOVA. ^*^ *p*＜0.05.
